# Essential Oils in Combination and Their Antimicrobial Properties 

**DOI:** 10.3390/molecules17043989

**Published:** 2012-04-02

**Authors:** Imaël Henri Nestor Bassolé, H. Rodolfo Juliani

**Affiliations:** 1 Laboratoire BAEBIB, UFR-SVT, Université de Ouagadougou, 09 BP 848 Ouagadougou, Burkina Faso; 2 Department of Plant Biology and Pathology, Rutgers, The State University of New Jersey, 59 Dudley Road, Foran Hall, New Brunswick, NJ 08901, USA; Email: hjuliani@rci.rutgers.edu

**Keywords:** essential oils, combinations, antimicrobial, synergism, antagonism, additive effects

## Abstract

Essential oils (EOs) have been long recognized for their antibacterial, antifungal, antiviral, insecticidal and antioxidant properties. They are widely used in medicine and the food industry for these purposes. The increased interest in alternative natural substances is driving the research community to find new uses and applications of these substances. EOs and their components show promising activities against many food-borne pathogens and spoilage microorganisms when tested *in vitro*. In food systems, higher concentrations of EOs are needed to exert similar antibacterial effects as those obtained in *in vitro* assays. The use of combinations of EOs and their isolated components are thus new approaches to increase the efficacy of EOs in foods, taking advantage of their synergistic and additive effects. The purpose of this review is to provide an overview on the antimicrobial efficacy of these combinations. A survey of the methods used for the determination of the interactions and mechanisms involved in the antimicrobial activities of these combinations are also reported.

## 1. Introduction

Plants produce a high diversity of secondary metabolites with a prominent function of protecting plants against predators and microbial pathogens due to their biocidal properties against microbes or repellence to herbivores. Some metabolites are also involved in defense mechanisms against abiotic stress (e.g., UV-B exposure) and are important in the interaction of plants with other organisms (e.g., attraction of pollinators) [1,2]. It is believed that most of the 100,000 known secondary metabolites are involved in plant chemical defense systems, they seemed to have appeared as a response of plants to the interactions with predators throughout the millions of years of co-evolution. There are three major groups of secondary metabolites, including terpenes, phenylpropenoids and N- and S-containing compounds [3]. Among these secondary metabolites, it is estimated that over 3,000 essential oils (EOs) are known, of which about 300 are commercially important and used by the flavor and fragrance industries [4]. 

Essential oils, or aromatic plant essences, are volatile and fragrant substances with an oily consistency typically produced by plants. They can be liquid at room temperature though a few of them are solid or resinous, and showing different colors ranging from pale yellow to emerald green and from blue to dark brownish red [5]. They are synthesized by all plant organs, *i.e.*, buds, flowers, leaves, stems, twigs, seeds, fruits, roots, wood or bark, and are stored in secretory cells, cavities, canals, epidermic cells or glandular trichomes [6]. Several techniques can be used to extract EOs from different parts of the aromatic plant, including water or steam distillation, solvent extraction, expression under pressure, supercritical fluid and subcritical water extractions.

The term “essential oil” was used for the first time in the 16th century by Paracelsus von Hohenheim, who referred to the effective component of a drug as “Quinta essential” [7]. The first reference on the uses of EOs for therapeutic reasons was found in the Ebers papyrus. This document listed in detail more than 800 EOs remedies and treatments and showed that myrrh was a favorite ingredient, often mixed with honey and other herbs, because of its ability to inhibit bacterial growth. The first bactericidal experiment of EOs is said to have been carried out by de la Croix in 1881 [8]. However, since those times the use of EOs in medicine gradually decreased as their use as flavor and fragrances increased [9].

Essential oils, also known as volatile oils, are complex mixtures of volatile constituents biosynthesized by plants, which mainly include two biosynthetically related groups [10]. These main groups include terpenes and terpenoids and aromatic and aliphatic constituents, all characterized by low molecular weight. 

Most of the antimicrobial activity in EOs is found in the oxygenated terpenoids (e.g., alcohols and phenolic terpenes), while some hydrocarbons also exhibit antimicrobial effects [11,12,13]. Interactions between these components may lead to antagonistic, additive or synergistic effects. Some studies have demonstrated that whole EOs usually have higher antibacterial activity than the mixtures of their major components, suggesting that the minor components are critical to the synergistic activity, though antagonistic and additive effects have also been observed [14,15,16].

Usually combinations, either single EOs or artificial mixtures of purified main components, affect multiple biochemical processes in the bacteria, producing a plethora of interactive antibacterial effects [13,17]. In recent years, there has been an increased interest in the use of natural antimicrobial agents thus the use of these combinations are strategies to control food-borne bacteria and other pathogenic microorganisms [13,18,19,20]. In view of these findings, the aim of this contribution is to review and highlight the antimicrobial efficacy of these combinations, and to provide the methods to determine the type of interactions and the mechanisms involved in the antimicrobial activities of these combinations.

## 2. Interaction between Components of Essential Oils

The antimicrobial properties of EOs have been reported in several studies [6,11,21]. In many cases the activity results from the complex interaction between the different classes of compounds such as phenols, aldehydes, ketones, alcohols, esters, ethers or hydrocarbons found in EOs [11,22,23]. Though in some cases, the bioactivities of EOs are closely related with the activity of the main components of the oils [24]. Several studies have found that a number of these compounds exhibited significant antimicrobial properties when tested separately [21,25,26,27,28,29,30].

It has been reported that EOs containing aldehydes or phenols, such as cinnamaldehyde, citral, carvacrol, eugenol or thymol as major components showed the highest antibacterial activity, followed by EOs containing terpene alcohols. Other EOs, containing ketones or esters, such as β-myrcene, α-thujone or geranyl acetate had much weaker activity. While volatile oils containing terpene hydrocarbons were usually inactive [28,31,32,33,34,35,36,37,38].

High antimicrobial activity of *Thymus* and *Origanum* species has been attributed to their phenolic components such as thymol and carvacrol [23,26,39,40,41] and those of *Eugenia caryophillus* [38], *Syzygium aromaticum* [42,43,44], *Ocimum basilicum* [30] to eugenol. The antimicrobial activity of the EO of *Cinnamomum zeylanicum* has been related to its cinnamaldehyde content [45], though cinnamaldehyde-containing oils (non-phenolic) showed lower antimicrobial activities than eugenol oils [44]. In basil, the strongest antimicrobial activity of sweet basil was attributed to eugenol (19%) and linalool (54%) content and a synergistic effect was observed. The importance of the hydroxyl group (-OH) of phenols was demonstrated by the higher antimicrobial and antioxidant activities of eugenol in relation to methyl eugenol (-O-Me) [46]. Terpinen-4-ol is considered to be the principal active component of *Melaleuca alternifolia* (tea tree) oil [34,35,47]. Lis-Balchin and Deans [48] showed that EOs containing large amounts of 1,8-cineole were better anti-listerial agents than EOs devoid of it. The weak antimicrobial activity of the EOs of *Chaerophyllum libanoticum* [49], *Tanacetum argenteum *subsp*. ﬂabellifolium* [50], *Cupressus arizonica* [51] has been attributed to their high hydrocarbon content.

Different terpenoid components of EOs can interact to either reduce or increase antimicrobial efficacy [13]. The interaction between EO compounds can produce four possible types of effects: indifferent, additive, antagonistic, or synergistic effects [11,52,53,54]. An additive effect is observed when the combined effect is equal to the sum of the individual effects. Antagonism is observed when the effect of one or both compounds is less when they are applied together than when individually applied. Synergism is observed when the effect of the combined substances is greater than the sum of the individual effects [11] while the absence of interaction is defined as indifference.

Interestingly, phenolic monoterpenes and phenylpropanoids (typically showing strong antimicrobial activities) in combination with other components were found to increase the bioactivities of these mixtures. Most of the studies have focused on the interaction of phenolic monoterpenes (thymol, carvacrol) and phenylpropanoids (eugenol) with other groups of components, particularly with other phenols, phenylpropanoids and monoterpenes alcohols, while monoterpenes and sesquiterpenes hydrocarbons were used to a lesser extent (Table 1). The combination of phenolics with monoterpenes alcohols produced synergistic effects on several microorganisms, in particular, the combination of phenolics (thymol with carvacrol, and both components with eugenol) were synergistically active against *E. coli *strains. Though other reports have observed additive [23] and antagonism effects [55] (Table 1). 

**Table 1:** Combination of components and essential oils and their antimicrobial interactions against several microorganisms.

Pair combinations	Organism	Methods	Interaction	References
Thymol/carvacrol	*Staphylococcus Aureus*, *Pseudomonas. Aeruginosa *	Half dilution	Additive	Lambert *et al. *[23]
	*Escherichia Coli *	Checkerboard	Synergism	Pei *et al. *[54]
	*S. aureus*, *Bacillus. cereus*, *E coli*	Checkerboard	Antagonism	Gallucci *et al. *[55]
	*S. aureus*, *P. aeruginosa*	Mixture	Additive	Lambert *et al. *[23]
	*E. coli *	Checkerboard	Additive	Rivas *et al*. [56]
	*Salmonella typhinurium *	Mixture	Synergism	Zhou *et al. *[57]
Thymol/eugenol	*E. coli *	Checkerboard	Synergism	Pei *et al. *[54]
Carvacrol/eugenol	*E. coli *	Checkerboard	Synergism	Pei *et al. *[54]
	*S. aureus*,*B. cereus*, *E coli*	Checkerboard	Antagonism	Gallucci *et al. *[55]
Carvacrol/myrcene	*S. aureus*, *B. cereus*, *E coli*	Checkerboard	Antagonism	Gallucci *et al.* [55]
Carvacrol/Cymene	*B. cereus*	Mixture	Synergism	Ultee *et al.* [58]
Carvacrol/linaloolEugenol/linaloolEugenol/menthol	*Listeria monocytogenes*, *Enterobacter aerogenes*,*E. coli*,*P. aeruginosa*	Checkerboard	Synergism	Bassole *et al.* [30]
Menthol/GeraniolMenthol/Thymol	*S. aureus*, *B. cereus*		Synergism	Gallucci *et al.* [55]
Cinnamaldehyde/Carvacrol	*E. coli *	Checkerboard	Additive	Pei *et al.* [54]
	*S. typhinurium *	Mixture	Synergism	Zhou *et al.* [57]
Cinnamaldehyde/Thymol	*E. coli *	Checkerboard	Synergism	Pei *et al.* [54]
	*S. typhinurium *	Mixture	Synergism	Zhou *et al.* [57]
Cinnamaldehyde/Eugenol	*Staphylococcus sp.*,*Micrococcus sp.*, *Bacillussp.*,*and Enterobacter sp.*	Mixture	Additive	Moleyar et Narasimham [59]
1,8-Cineole/Aromadendrene	*methicillin-resistant S. aureus (MRSA) and vancomycin-resistant enterococci (VRE) Enterococcus faecalis*	Checkerboard	Additive	Mulyaningsih *et al.* [60]
Limonene/1,8-cineole	*S. aureus*, *P. aeruginosa*	Mixture	Synergism	van Vuuren and Viljoen [61]
α-pinene/Limonene	*Saccharomyces cerevisiae*	Checkerboard	synergism, additive	Tserennadmid *et al.* [62]
α-pinene/Linalool				
Linalool/Terpinen-4-ol				
*O. vulgare/Rosmarinus ofﬁcinalis*	*L. monocytogenes*, *Yersinia enterocolitica*, *Aeromonas hydrophilla*, *P. ﬂuorescens*	Mixture	synergism	de Azeredo *et al. *[63]
*O. vulgare/T. vulgaris*	*P. ﬂuorescens*	Mixture	Additive	
*Lippia multiflora/Mentha piperita*	*E. coli*, *E. aerogenes*, *Enterococcus faecalis*,*L. monocytogenes*, *P. aeruginosa*, *Salmonella enterica*, *S. typhimurium, Shigella. dysenteriae*, *S*. *Aureus*	Checkerboard	Synergism, additive	Bassole *et al. *[30]
*L. multiflora/O. basilicum*				
*M. piperita/O. basilicum*	*E. coli*, *E. aerogenes*, *E. faecalis*, *L. monocytogenes*, *P. aeruginosa*, *S. enterica*,*S. typhimurium*, *S. dysenteriae*, *S. aureus*			
*S. aromaticum/R. ofﬁcinalis*	*Staphylococcus. epidermidis*, *S aureus*, *B. subtilis*, *E. coli*, *Proteus vulgaris*, *P. aeruginosa*	Mixture	Additive	Fu *et al. *[42]
	*Candida albicans*		Synergism	
	*Aspergillus niger*		Antagonism	
*C. zeylanicum/S. aromaticum*	*E. coli*	Mixture	Antagonism	Goni *et al.* [64]
	*Y. enterocolitica*, *L. monocytogenes, B. Cereus*	Mixture	Synergism	
*O. vulgare/O. basilicum*	*B. Cereus*, *E. Coli*,*P. Aeruginosa *	Checkerboard	Additive	Gutierrez *et al.* [20]
*O. vulgare/Melissa ofﬁcinalis*	*B. cereus *			
*O. vulgare/O. majorana*	*B. cereus*,*E. coli *			
*O. vulgare/R. ofﬁcinalis*	*B. cereus *			
*O. vulgare/T. vulgaris*	*Enterobacter cloacae*,*P. ﬂuorescens*,*Listeria Innocua *	Checkerboard	Additive	Gutierrez *et al.* [65]
*O. vulgare/Salvia triloba*	*B. cereus *			
*O. vulgare/T. vulgaris*	*B. cereus*,*P. aeruginosa *			
*O. vulgare/T. vulgaris*	*Enterobacter cloacae*,*P. ﬂuorescens*,*Listeria Innocua *	Checkerboard	Additive	Gutierrez *et al.* [65]
*T. vulgaris/O. majorana*	*E. cloacae *			
*T. vulgaris/M. ofﬁcinalis*	*L. innocua *			
*Cymbopogon citratus/C. giganteus*	*E. coli*, *E. aerogenes*, *L. monocytogenes*, *S. typhimurium*, *S. dysenteriae*, *S. aureus*	Checkerboard	Synergism, additive	Bassole *et al.* [66]

Mixtures of cinnamaldehyde with carvacrol or thymol yielded in most cases synergistic effects against *E. coli* and *S. typhinurium*, though in one case an additive effect was observed (Table 1). Other monoterpenes have also been tested, particularly the oxide 1,8-cineole that in combination with sesquiterpene and monoterpene hydrocarbons (e.g., aromadendrene and limonene) were found to have additive and synergistic effects, respectively. Other combinations including a monoterpene hydrocarbon (α-pinene) with limonene or linalool also showed additive and synergistic effects (Table 1).

Mixture of EOs have also been shown to interact with each other acting as additive, synergistic and in a few cases antagonistic agents (Table 1). The essential oil of oregano (*Origanum vulgare*) was the most used EO (rich in thymol and carvacrol) and combined with rosemary (*Rosmarinus officinalis*), thyme (*Thymus vulgaris*), basil (*Ocimum basilicum*), marjoram (*O. majorana*) and lemon balm (*Melissa officinalis*) (Table 1). In most cases only additive effects were observed, only the combination with rosemary oil yielded synergistic effects (Table 1).

Most studies attributed additive and synergism effects to phenolic and alcohol compounds (Table 1). Generally compounds with similar structures exhibit additive rather than synergistic effect. The occurrence of additive interaction of some essential oils has been related to their main phenolic compounds (carvacrol and thymol) [21,23,63]. Antagonistic effect has been attributed to the interaction between non-oxygenated and oxygenated monoterpene hydrocarbons [25,64].

## 3. Interaction Test Methods

The assessment of the interaction between essential oil components is based on using macro- or micro-dilution techniques. Checkerboard, Graphical and Time-kill methods are the most widely used procedures. The principles and practice of these methods are described in the literature. These methods were preliminary developed for the detection of drug synergism thus there is no standardized method developed for evaluating the interaction between essential oils or their components [67,68,69].

The checkerboard test requires determination of the fractional inhibitory concentration (FIC) or the effect of the combination index (EC index) of each agent [54,57,70,71,72]. The FIC of a factor is the concentration that kills when used in combination with another agent divided by the concentration that has the same effect when used alone [73,74]. Generally, minimum inhibitory concentrations (MICs) or concentrations of maximal inhibition (C_max_) are used as the reference concentrations [13,30,55,60,62,64]. The FIC index for the combination of A and B is the sum of their individual FIC values. Each checkerboard test generates many different combinations, and by convention, the FIC values of the most effective combination are used in calculating the FIC index. The FIC index defines the nature of the interaction. The values of the FIC index used for the definition of the nature of the interaction differs between publications and makes comparison between studies difficult (Table 2). The definition of the reference concentration differs between publications and this is another obstacle at the time of comparing different research studies [11,54,64].

**Table 2:** Fractional inhibitory concentration (FIC) index used to determine the type of interaction.

FIC index				References
Synergy	Addition	Indifference	Antagonism	
<1	1	1–2	>2	Pei *et al.* [54]
<0.5	0.5–1	1–4	>4	Schelz *et al.* [75], Gutierrez *et al.* [20,65], Bassole *et al.* [30,66]; Tserennadmid *et al.* [62]
≤0.5	0.5–1	1–4	>4	Mulyaningsih *et al.* [60]
<0.5	0.5–4	-	>4	Zore *et al.* [76]; Goni *et al.* [64]
≤0.5	0.5–1	-	>1	Rosato *et al.* [77]
≤0.75	0.75-2	–	>2	Galluci *et al.* [55]
<0.9	0.5–1,1	-	1.1	Romano *et al.* [78]

The effect of the combination index (EC index) is the absolute value of the difference between logarithms of the difference in population (DP) in the combination system and the single agent, respectively [54,57]. This index is used to determine synergistic effect of a combination on the basis on three principles:

(1) The decrease in populations (DP > 90%): linked with the definition of DP, it was concluded that only when DP < 0.1 (log DP < −1) that the combinations of various reagents had significant antibacterial activity.
(2) When there was significant difference (ANOVA) between the antibacterial activity of the combination and the individual components, respectively, it meant that the combination was effective.
(3) Synergy was defined as a 2-log decrease of Colony Forming Units (CFU) in the drug combination group compared with the most effective single agent at the end of 24 h [79].

Some authors have mentioned that the results of the checkerboard assay can be represented graphically by plotting the FIC values on a graph known as an isobologram [73,74]. On the x- and y-intercepts the half maximal effective concentration (EC_50_) are plotted or MIC values of the two agents when used alone [80]. Additive effects will produce straight lines while synergy will produce a concave curve and antagonistic effects a convex one [80]. 

The isobolograms of *Salvia chamelaeagnea L.* and *Leonotis leonurus L.* at various ratios against four pathogens showed that synergistic interactions were obtained against Gram-positive bacteria for nearly all ratios, while mostly antagonistic or additive interactions were observed with Gram-negative bacteria [81]. Delaquis *et al.* [13] defined synergistic effects of mixed fractions of dill, cilantro, coriander, and eucalyptus EOs when the isobologram showed concave shape.

The time-kill method evaluates combined antimicrobial action by measuring the effect of a subinhibitory concentration of one agent on the killing ability of another over time [74]. A synergistic interaction is observed when the killing ability of the first agent is increased by a sub-inhibitory concentration of the second agent. An antagonistic interaction is present if the antimicrobial effect of the first component is inhibited by the second. Because one agent is used at sub-inhibitory concentrations, this assay cannot distinguish additive interactions (combined activity equals the sum of individual activities) from indifferent interactions [80]. Mulyaningsih *et al. *[60] reported additive and synergistic effects of the combinations of 1,8-cineole and aromadendrene against methicillin-resistant *Staphylococcus aureus* (MRSA) and vancomycin-resistant enterococci (VRE) and *Enterococcus faecalis* by using checkerboard and time-kill assays respectively. In addition to these methods, others have been reported. Fyfe *et al.* [82] considered the combined effects of plant volatile oils and benzoic acid derivatives against *L. monocytogenes* and *S. enteritidis* as synergistic when the combined components demonstrated ≥log10 higher inhibition than the sum of the inhibitory effects of the components used alone. Fu *et al. *[42] observed increased antifungal effects caused by combinations (1:5, 1:7 and 1:9) of essential oils of *S. aromaticum* (clove) and *R. ofﬁcinalis* against *C. albicans*. Lambert *et al. *[23] reported that carvacrol and thymol in combination showed additive effects against *S. aureus* and *P. aeruginosa* by using half-fold dilutions within the Bioscreen plate.

**Table 3:** Ratio of combined compounds and percentage of effective reduction concentration by synergy as compared with the individual components.

Pair synergistic combinations	Organisms	Ratio of combined compounds	Reduction of effective concentration (%)	References
Cinnamaldehyde/ Thymol	*E. coli*	1:1	25	Pei *et al.* [54]
Cinnamaldehyde/ Eugenol		1:4 or 1:8	50	
Thymol/carvacrol		1:1	25	
Thymol/Eugenol		1:4	50	
Carvacrol/Eugenol		1:4 or 1:8	25	
Geraniol/menthol	*S. aureus*		50	Gallucci *et al. * [55]
Thymol/eugenol	*B. cereus*		25	
Eugenol/geraniol			35	
Thymol/menthol			65	
Geraniol/menthol			94	
Cinnamaldehyde/Thymol	*S. typhinurium*	1:1	25	Zhou *et al.* [57]
Cinnamaldehyde/Carvacrol		1:1	25	
Thymol/carvacrol		1:1	50	
1,8-cineole/(+)-Limonene	*S. aureus*	9:1, 8:2, 7:3, 6:4		van Vuuren and Viljoen [61]
1,8-cineole/(±)-limonene	*P. Aeruginosa*	9:1, 8:2, 7:3, 6:4, 5:5, 4:6, 3:7, 2:8, 1:9		
(+)limonene/(-)limonene	*M. catarrhalis*	1:1	60	
α-pinene/limonene	*S. cerevisiae*		96	Tserennadmid *et al.* [62]
*O. vulgare/Rosmarinus ofﬁcinalis*	*L. monocytogenes* ,	1:16	50	de Azeredo *et al.* [63]
	*Yersinia enterocolitica* ,	1:16		
	*Aeromonas hydrophilla*	1:16		
	*P. * *ﬂ* *uorescens*	1:8		
	*L. monocytogenes*	2:1	90	
	*S. typhimurium*	2:1	90	
	*S. aureus*	1:2	80	
*Lippia multiflora/Mentha piperita*	*E. faecalis*	5:3	91	Bassole *et al. * [30]
	*L. monocytogenes*	8:1	86	
	*E. coli CIP*	16:1	81	
*M. piperita/O.basilicum*	*E. faecalis*	3:25	63	
	*L. monocytogenes*	3:25	73	
	*S. thyphimirium*	1:1	31	
	*S. dysenteria*	3:25	65	
	*S. aureus*	3:25	64	
*S. aromaticum/R. ofﬁcinalis*	*C. albicans*	1:5, 1:7; 1:9	-	Fu *et al.* [42]
*C. zeylanicum/S. aromaticum*	*Y. enterocolitica* ,	-	80	Goni *et al. * [64]
	*L. monocytogenes*	-	60	
	*B. cereus*	-	50	
*C. citratus/C. giganteus*	*E. aerogenes*	2:1	60	Bassole *et al.* [66]

However, due to the diversity of the methods, there is no an effective and standardized way to evaluate and quantify the synergistic effects of EOs combination, making it impossible to compare the results of the reports. Besides, the concentrations or ratios of the mixtures are not always provided by the authors so comparisons are not always possible (Table 1 and Table 3).

## 4. Mechanism of Action

There are fewer reports on the mechanisms of action of combination of essential oils or their purified components on microorganisms [11,83,84,85,86]. Some publications deal with the mode of action of the essential oil components in combination with other natural preservatives or antibiotics [58,87,88,89,90,91,92,93,94,95,96,97,98,99,100]. There are limited numbers of papers dealing with the mechanism of action of combinations of the essential oils or their components. However, there are some generally accepted mechanisms of antimicrobial interaction that produce synergism. They include the sequential inhibition of a common biochemical pathway, inhibition of protective enzymes and use of cell wall active agents to enhance the uptake of other antimicrobials [19]. Synergism between carvacrol and some hydrocarbons monoterpenes (such as α-pinene, camphene, myrcene, α-terpinene and *p*-cymene) that typically showed low antimicrobial properties has been observed [58,63]. The capacity of hydrocarbons to interact with cell membrane facilitates the penetration of carvacrol into the cell [58,63,101]. Pei *et al.* [54] hypothesized that the synergistic effects of eugenol/carvacrol and eugenol/thymol might be due to the fact that carvacrol and thymol disintegrated the outer membrane of *E. coli*, making it easier for eugenol to enter the cytoplasm and combine with proteins. It was also observed the synergistic effect of eugenol/cinnamaldehyde is probable due to the interaction of these components with different proteins or enzymes. 

The combination of pair of components showing synergistic effects will then reduce the concentration needed to yield the same microbial effect when compared with the sum of the purified components. Thus, the synergistic effects of cinnamaldehyde and thymol against *E. coli* had an effective reduction of concentration of 25% (Table 3), similar reduction at the same ratio was observed for *T. typhinurium*. The combination of cinnamaldehyde and reduced levels of eugenol generated a 50% reduction of the concentration. In the case of cinnamaldehyde and thymol the working ratio was of 1:1, while in the case of cinnamaldehyde and eugenol, lower levels of eugenol (1:4–1:8) were needed to reduce the concentration. Thymol and carvacrol at ratio of 1 to 1 also showed similar results (reduction of 25%), the use of thymo and eugenol at 1 to 4 further reduced the concentration to 50%. In the pair carvacrol/eugenol, the same ratios of 1 to 4 showed a reduction of 25%.

Zhou *et al. *[76] proposed two hypotheses to explain synergistic effects of cinnamaldehyde/thymol or cinnamaldehyde/carvacrol against *S. typhimurium*:

- Thymol or carvacrol could increase the permeability of the cytoplasmic membrane, and probably enable cinnamaldehyde to be more easily transported into the cell.
- Thymol or carvacrol could increase the number, size or duration of existence of the pores created by the binding of cinnamaldehyde to proteins in the cell membrane, so that a synergistic effect is achieved when these two components are used in combination.

These authors proposed three hypothesis that could explain the synergistic effect between thymol/carvacrol against *S. typhimurium*: (a) the antibacterial mechanism of thymol and carvacrol might be different; they act on the different targets of *S. typhimurium*; (b) the synergistic effect could be due to the similarity of their mechanism; and (c) the synergistic effect occurs only when they inhibit together *S. typhimurium*. More recently, Fei *et al*. [102] showed that the synergistic combinations of EOs of oregano/basil against *E. coli*, basil/bergamot against *S. aureus*, oregano/bergamot against *B. subtilis* and oregano/perilla against *S. cerevisiae* significantly disrupted the integrity of cell membranes when compared with control untreated membranes.

The practical implications of these observations are important at the time of using EOs components in food systems since the use of the lower concentration needed to yield a similar antibacterial activity will mean reduced flavor notes in foods products (Table 3). For certain foods, some EO components in high concentrations can impart undesirable notes to foods (e.g., eugenol).

Mechanisms of interaction that produced antagonistic effects were less studied. Some of the studies included combinations of bactericidal and bacteriostatic agents, use of compounds that act on the same target of the microorganism and chemical (direct or indirect) interactions among compounds such as the reduction of the active aqueous terpene solubility by non-aqueous monoterpene hydrocarbons [26,64]. 

## 5. Other Factors Affecting the Interaction of Components

There are limited number of studies on the effects of the test medium physical and chemical parameters on the interaction between essential oil components and their antimicrobial activities. Physical (temperature) and chemical (sodium chloride) parameters were also found to modulate the antimicrobial responses of the mixtures. Sodium chloride was found to have antagonistic effects when combined with carvacrol and *p*-cymene against *B. cereus*. It was also observed that carvacrol and p-cymene worked synergistically, but this effect was reduced when sodium chloride was added (1.25 g/L) [58]. It has been reported that the combination of cinnamon and clove EOs showed better antimicrobial activity in vapor phase than in liquid phase [64]. In the study of the combined effects of thymol, carvacrol and temperature on the quality of non conventional poultry patties by using a simplex centroid mixture design, the best effects were obtained when the patties were mixed with both compounds and stored at low temperature 0 to 3 °C [103].

## 6. Conclusions

Due to the limited number of studies and in order to optimize the synergy potential of mixtures, research should focus on: (a) the effects of intrinsic and extrinsic parameters of test medium (pH, fat, protein, water content, incubation time/temperature, packaging procedure, and physical structure) on the combinations of essential oils or their components and their antimicrobial properties; (b) the mechanism of action of the synergisms, additions or antagonisms to optimize the activity in food preservation, medicine and cosmetic; (c) possible toxicity of combined essential oils or components; (d) development of standardized methods for the evaluation of the interaction between essential oils or their components.

Essential oils are natural plant products containing complex mixture of components and thus having multiple antimicrobial properties. Most of the antimicrobial activity in EOs appears to derive from oxygenated terpenoids, particularly phenolic terpenes, phenylpropanoids and alcohols. Other constituents (e.g., hydrocarbons) that tipically showed low activities can be used in combinations to increase their bioactivities. Interactions between these components may lead to antagonistic, additive or synergistic effects. Checkerboard, graphical and Time-kill methods are the most widely used procedures to assess of the interaction of essential oil components. Investigations should be carried out on their mode of action and their probable toxicological effects in order to optimize their use.
